# Fast-growing growth hormone transgenic coho salmon (*Oncorhynchus kisutch*) show a lower incidence of vaterite deposition and malformations in sagittal otoliths

**DOI:** 10.1242/jeb.244099

**Published:** 2022-10-10

**Authors:** Irvin Chalan, Laia Solsona, Clara Coll-Lladó, Paul B. Webb, Dionne Sakhrani, Robert H. Devlin, Daniel Garcia de la serrana

**Affiliations:** ^1^ Cell Biology, Physiology, and Immunology Department, School of Biology, University of Barcelona, 643 08028 Barcelona, Catalonia, Spain; ^2^ School of Chemistry, University of St Andrews, St Andrews KY16 9ST, UK; ^3^ Fisheries and Oceans Canada, 4160 Marine Drive, West Vancouver, BC, Canada, V7V 1N6

**Keywords:** Otolith, Salmon, Transgenic, Vaterite

## Abstract

In fish otoliths, CaCO_3_ normally precipitates as aragonite, and more rarely as vaterite or calcite. A higher incidence of vaterite deposition in otoliths from aquaculture-reared fish has been reported and it is thought that high growth rates under farming conditions might promote its deposition. To test this hypothesis, otoliths from growth hormone (GH) transgenic coho salmon and non-transgenic fish of matching size were compared. Once morphometric parameters were normalized by animal length, we found that transgenic fish otoliths were smaller (−24%, −19%, −20% and −30% for length, width, perimeter and area, respectively; *P*<0.001) and rounder (−12%, +13.5%, +15% and −15.5% in circularity, form factor, roundness and ellipticity; *P*<0.001) than otoliths from non-transgenic fish of matching size. Interestingly, transgenic fish had smaller eyes (−30% eye diameter) and showed a strong correlation between eye and otolith size. We also found that the percentage of otoliths showing vaterite deposition was significantly smaller in transgenic fish (21–28%) than in non-transgenic fish (69%; *P*<0.001). Likewise, the area affected by vaterite deposition within individual otoliths was reduced in transgenic fish (21–26%) compared with non-transgenic fish (42.5%; *P*<0.001). Our results suggest that high growth rates per se are not sufficient to cause vaterite deposition in all cases, and that GH overexpression might have a protective role against vaterite deposition, a hypothesis that needs further investigation.

## Teaser

**Summary:** Coho salmon transgenic for growth hormone have lower laterite incidence and malformations.

## BACKGROUND

Otoliths, also known as ear stones, are located in the fish inner ear and are responsible for animal's hearing, balance and navigation (Popper et al., 2005). There are three pairs of otoliths of different sizes, the sagitta (located in the saccule), lapillus (located in the utricle) and asteriscus (located in the lagena). In a great number of fish families, the sagitta is the largest otolith, followed by the lapillus and the asteriscus, with some exceptions such as the otophysan fishes, where the lapilli and asterisci can be the largest otoliths. Otoliths are formed by deposition of calcium carbonate (CaCO_3_) on an organic protein matrix, a process controlled by the surrounding endolymph composition (Payan et al., 2004) and influenced by both environmental (e.g. temperature or pH) and physiological factors (e.g. nutritional status, sexual maturation, stress, age and endocrine status) (Esbaugh et al., 2012; Fablet et al., 2011; Hüssy, 2008; Mu et al., 2018; Stormer and Juanes, 2016). The CaCO_3_ in biological systems can crystalize in three different CaCO_3_ polymorphs: aragonite, vaterite and calcite. Except for some primitive species of fish (Pracheil et al., 2017), the sagitta and lapillus are generally formed by aragonite while the asteriscus is commonly formed by vaterite (MacDonald et al., 2012). Vaterite depositions can also be found in sagitta and lapillus otoliths in around 1–24% of wild populations, with calcite being much rarer (Hughes et al., 2004; Nehrke et al., 2012). Otoliths can start depositing CaCO_3_ as aragonite and then later switch to other polymorphs such as vaterite, a change that seems to be irreversible (Reimer et al., 2017). Vaterite deposition is commonly found in a range of fishes reared under aquaculture conditions, being up to 3.7 times more frequent compared with wild populations (David et al., 1994; Tomas and Geffen, 2003). However, this proportion is much higher in the case of salmonids, with some studies reporting 80–100% of the individuals showing some degree of vaterite deposition (Gauldie, 1986; Reimer et al., 2016; Sweeting et al., 2004). Vaterite otoliths are larger, more translucent, more fragile and more irregular than those formed by aragonite. In addition, it has been observed that vaterite has a significant effect on the transmission of sound waves, with up to 50% loss of hearing, fish navigation capacities (Reimer et al., 2016) and escape kinematics (Vignon and Aymes, 2020). These effects are detrimental to the well-being of fish and the development of their natural behaviour (such as migration). Therefore, understanding why some farmed animals replace aragonite with vaterite is important for improving animal welfare and survival. The mechanisms by which any specific CaCO_3_ polymorph is deposited in the otoliths were quite elusive for many years. However, in the last decade, research focused on the otolith protein matrix has unravelled the role of the constituent proteins mediating CaCO_3_ crystal formation. The early discovery of some of the main matrix proteins such as otolith matrix protein 1 (Omp-1) (Murayama et al., 2005) and otolin-1 (Tohse et al., 2008) has led to a growing number of proteins being identified, such as starmaker (Stm), starmaker-like (Stm-l), otoconin (Otoc1), otolith matrix macromolecule 64 (Omm-64) and osteonectin (Sparc). Other studies have found that changes in the protein matrix phosphorylation status can also alter aragonite deposition (see review by Thomas et al., 2019).

It has been postulated that aquaculture rearing conditions (e.g. animal density, temperature fluctuation, continuous light treatment, food regime, water quality) may trigger the replacement of aragonite by vaterite as the main polymorph, but the specific mechanisms are not completely clear. Early studies ruled out the possibility of a genetic predisposition to replace aragonite by other CaCO_3_ polymorphs (Gauldie, 1986), but with better genotyping and pedigree reconstruction techniques, this idea has recently been re-examined (Coll-Lladó et al., 2018), indicating a genetic susceptibility to replace aragonite. In addition, Reimer et al. (2017) suggested that the probable cause of vaterite deposition in aquaculture-reared animals was the high growth rates experienced under intensive aquaculture conditions. The authors suggested two possible mechanisms for how fast growth can promote vaterite deposition: (1) by modifying the otolin-1/Omm-64 proportion of the otolith organic matrix, and/or (2) by a lower [Ca^+2^]/[CO_3_^−2^] ratio due to a higher transport of HCO_3_^−^ towards the endolymph arising from high-energy availability in diets for fast-growth animals. However, recent proteomic studies question the hypothesis that differences in HCO_3_^−^ concentration either side of the otolith are directly responsible for vaterite deposition because of the presence of carbonic anhydrases not only in the saccular epithelium but also in the endolymph itself (Thomas et al., 2019).

The role of growth rate in aragonite replacement by vaterite is a very intriguing hypothesis that would benefit from additional assessment. In the current study, we investigated the influence of growth rate on vaterite deposition using a growth hormone (GH) transgenic coho salmon (*Oncorhynchus kisutch*) model that overexpresses GH and drives high growth rates (Devlin et al., 2004). GH, secreted by the fish brain in non-transgenic fish and by all tissues in GH-transgenic salmon, promotes physiological and metabolic changes that lead to an increase in growth (Jönsson and Björnsson, 2002; Causey et al., 2019; Raven et al., 2008). Some of the effects are achieved by direct interaction with its receptors or by promoting the secretion of insulin-like growth factors (IGFs), strong pro-anabolic hormones that also promote growth (Fuentes et al., 2013).

GH-transgenic coho salmon display a much higher appetite than their non-transgenic counterparts and a more efficient use of energy, among other physiological changes (Devlin et al., 2020), which allow these animals to at least double the growth rates observed in non-transgenic individuals. Also, the GH in transgenic fish promotes the use of carbohydrates as a main source of energy, having a sparing effect on proteins, and therefore promoting growth (Higgs et al., 2009). In the present work, we used size-matched GH-transgenic coho salmon and non-transgenic coho salmon fed to satiation to study otolith growth, shape and crystallization. We hypothesized that if growth rates were (partly or totally) responsible of vaterite deposition, animals from the GH-transgenic group would have a higher incidence of vaterite in their otoliths compared with non-transgenic fish.

## MATERIALS AND METHODS

### Animal breeding, rearing and sampling

Two strains of coho salmon, *Oncorhynchus kisutch* (Walbaum 1792), were used in this study. Adult wild-type salmon were collected in the autumn of 2018 from the Chehalis River Hatchery (BC, Canada) and a random population of non-transgenic (NT) salmon was generated therefrom by crossing 10 single pairs and pooling their progeny. Coho salmon hemizygous for the OnMTGH1 growth hormone transgene (strain M77) (Devlin et al., 2004) were generated by crossing male salmon homozygous for the transgene with wild-type coho salmon also from the Chehalis River (as above). Rearing broods of salmon one year apart (with the same Chehalis River wild-type genetic background, and both fed to satiation) allowed matching of fish sizes (as measured by standard length) between the wild-type NT salmon and transgenic strains at a stage suitable for otolith isolation and analysis. In addition to the use of multiple parents from the same strain with the aim of reducing genetic effects, two batches of coho salmon (generated in 2018 and 2019) were used and animals from both batches were grown until NT and transgenic fish sizes matched. A group of transgenic animals was also allowed to grow for longer until their eye diameter matched that of the NT group [referred to as older transgenic fish (TG group) in figures and tables; see Discussion]. Fish were reared in aerated freshwater in 3000 l tanks with simulated natural lighting and photoperiod. Fish were fed to satiation 3 times a day with commercial salmon diets (Skretting Canada, Vancouver, BC, Canada).

All animal rearing, procedures and experimental protocols (see below) were conducted in compliance with the Canadian Council on Animal Care guidelines and by review and approval from the Fisheries and Oceans Canada Pacific Region Animal Care Committee under animal use protocol AUP 19-018A1. ARRIVE guidelines (Animal Research: Reporting of *In Vivo* Experiments; https://arriveguidelines.org) were also followed: the experimental groups (transgenic versus NT fish) and experimental units (individual animals) are specified, sample sizes are indicated, sample sizes were chosen to provide sufficient animals for regression analyses, fish were selected randomly from groups and none was excluded (hence no blinding for sample selection could arise), outcome measures were on the whole otolith characteristics derived from individual fish, statistical methods are described in Materials and Methods and were determined *a priori*, and summary statistics of body size measures are provided in the Results and Discussion. Fish were euthanized in 200 mg l^−1^ tricaine methanesulfonate buffered with 400 mg l^−1^ sodium bicarbonate as specified under the Fisheries and Oceans Canada Pacific Region Animal Care Committee animal use protocol AUP 19-018A1. Following death of the fish, standard length (from snout to the end of the last vertebra), eye diameter and head size (from snout to the end of the operculum) were measured. The head was severed from the body just posterior to the caudal edge of the operculum and immediately frozen at −70°C. All fish sampled were included in the analysis.

### Otolith extraction and measurements

Frozen heads were slowly thawed overnight in a 4°C fridge. Heads were opened with a pair of scissors, allowing the brain to be extracted with a spatula. With the help of a toothpick and fine forceps, we searched the inner ear cavity for the sagittal otoliths. The right and left sagitta were carefully extracted. Otoliths were carefully cleaned from any remaining tissue and stored in 1.5 ml Eppendorf tubes containing 500 µl of 70% ethanol. Otoliths were individually photographed against a black background and using homogeneous light an electric camera (Jiusion) with eight LEDs fixed to a support (RS2 copylizer, Kaiser Fototechnik) placed at the same position for each fish and including a millimetre scale for internal calibration.

Otolith length (OL), height (OH), area (OA) and perimeter (OP) were measured using ImageJ software (Schneider et al., 2012). To ensure the accuracy of the measurements, otoliths were measured twice by different people. For a better comparison between animals OL, OH, OP and OA were normalized against standard length and/or eye diameter, generating normalized OL, OH, OP and OA parameters (Table 1).

**Table 1.**
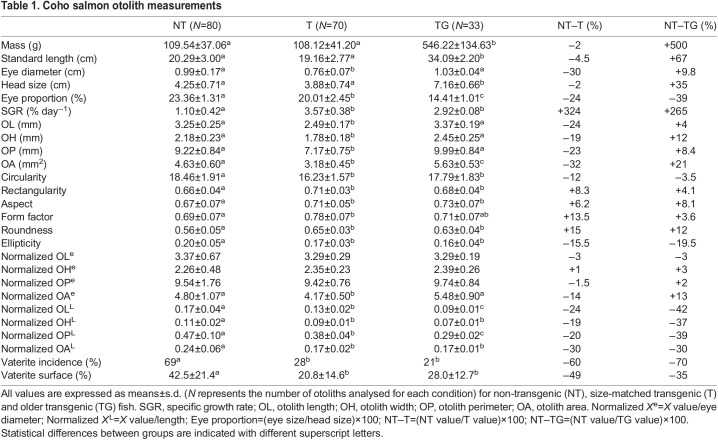
Coho salmon otolith measurements

Otolith shape indexes were estimated from otolith measurements as previously described (Leguá et al., 2013; Pothin et al., 2006):
(1)

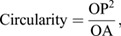

(2)

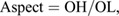

(3)

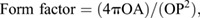

(4)

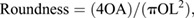

(5)

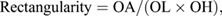

(6)

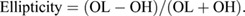
Otolith outlines from each group were estimated using the R-build program ShapeR (Libungan and Pálsson, 2015) with a smooth factor of 10.

Otolith opacity and homogeneity were determined by transforming otolith pictures to black and white and analysing the grayscale intensity using Plot Profile and Surface plot tools from ImageJ.

### Vaterite determination

Because of its crystal structure, vaterite is more transparent than aragonite (which is opaque). To identify vaterite deposition in the otoliths, sagittas were photographed under a light microscope with a darkfield diaphragm and ×4 magnification, which helps to better identify vaterite deposition. The percentage of area covered by vaterite was estimated using ImageJ.

To confirm that the abnormal mineralization found in the otoliths was vaterite, 5 otoliths per treatment were analysed by Raman spectrometry with a Horiba Jobin Y von LabRam HR instrument using 514 nm excitation wavelength and 50× magnification, long working distance objective. Laser intensity was attenuated using neutral density filters to prevent laser-induced transformation of the polymorph.

### Statistical analysis

All statistical analyses were conducted using R-Studio v.1.1.419 (https://www.rstudio.com/products/rstudio/). Otolith measurements (OL, OH, OP and OA), standard length, eye diameter (normalized OL, OH, OP and OA) and shape indexes (circularity, aspect, form factor, roundness, rectangularity and ellipticity) were analysed using linear mixed models (*lme4* R-package; Bates et al., 2015). Tukey *post hoc* analyses were performed in all cases to determine significant differences between the treatments.

Unless indicated otherwise, all values are shown as means±s.d. Overall, statistical differences in otolith shape were analysed with ShapeR as described by Libungan and Pálsson (2015).

All graphs were produced using the ggplot2 R-build package (Wickham, 2016). R-regression plots including 95% confidence intervals were estimated using the *geom_smooth (method=“lm”)* flag. Differences were considered significant when *P*-values were less than 0.05.

## RESULTS

**Fig. 1.**
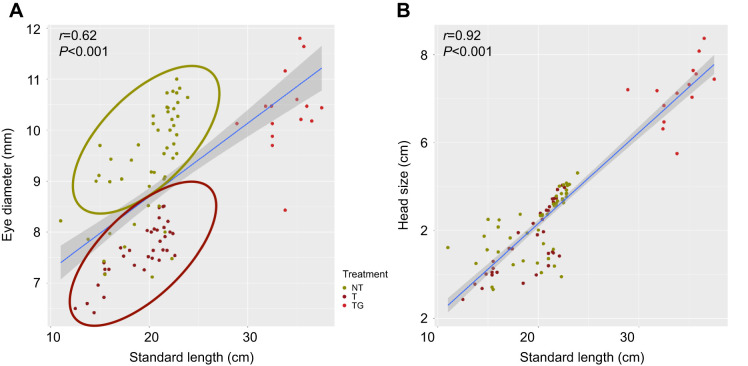
**Coho salmon body parameters.** Correlation between standard length and eye diameter (A) or head size (B) for non-transgenic (NT), size-matched transgenic (T) and older transgenic (TG) coho salmon. Notice that for animals of matching length, NT fish had a larger eye diameter (green ellipse) than transgenic fish (dark red ellipse). The shaded area around the linear correlation represents the 95% confidence interval of the correlation. Pearson coefficient of correlation (*r*) and degree of significance (*P*) are indicated.

A total of 40 transgenic and 36 NT coho salmon (*Oncorhynchus kisutch*) of matching standard length (19.16±2.77 and 20.29±3.00 mm, respectively; *P*=0.020) and body mass (108.12±41.20 and 109.54±37.06 g, respectively; *P*=0.36) (Tables 1 and 2) were initially analysed. Our data show that transgenic animals had significantly higher specific growth rate (3.57±0.38% day^−1^) than NT fish (1.10±0.42% day^−1^; *P*<0.001). We also found that transgenic individuals had significant smaller eyes (0.76±0.07 cm) than NT fish (0.99±0.17 cm; +30%; *P*<0.01), while head size was quite similar (only 2% smaller in transgenic animals) between treatments, with strong correlations between standard length against eye diameter (*r*=0.62; *P*<0.001) and head size (*r*=0.92; *P*<0.001) (Fig. 1).

**Table 2.**
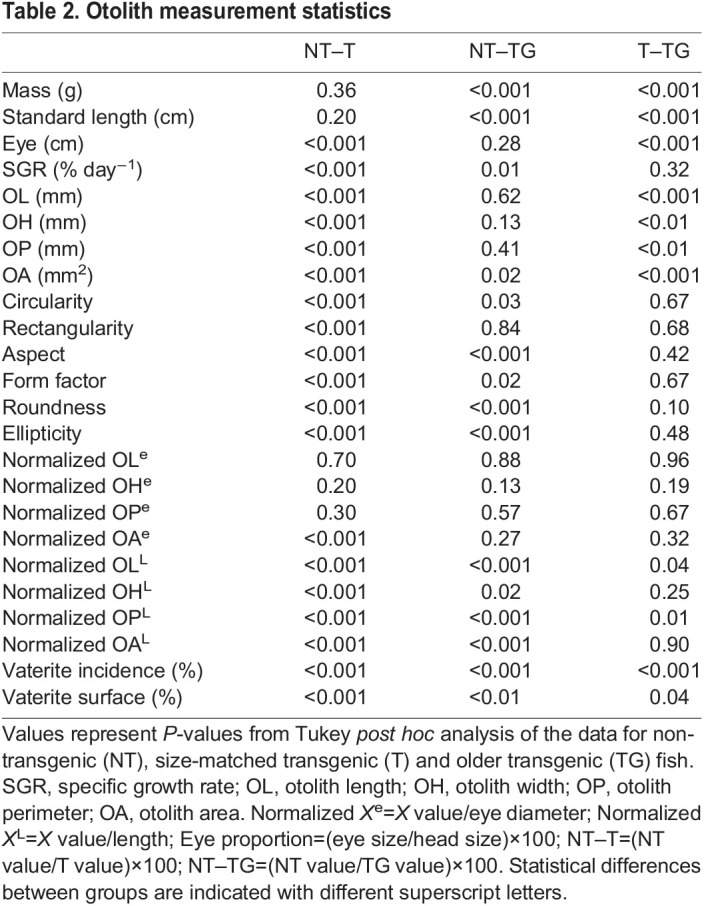
Otolith measurement statistics

**Fig. 2.**
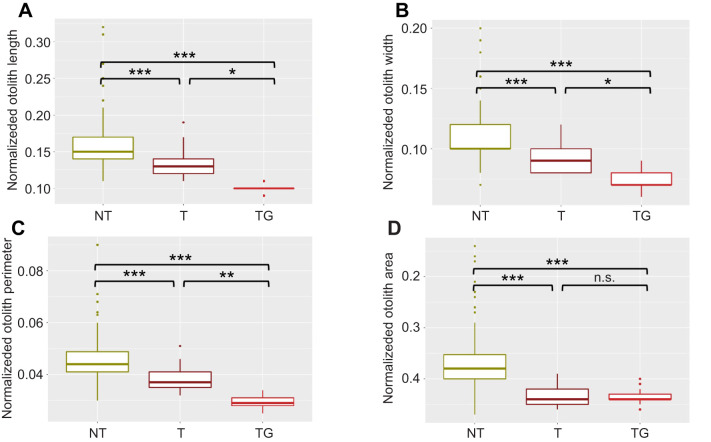
**Normalized otolith measurements.** Box and whisker plots (median is represented by the horizontal line, second and third quartiles by the rectangle areas and upper and lower quartiles as vertical lines) for normalized otolith length (A), width (B), perimeter (C) and area (D) for non-transgenic (NT), size-matched transgenic (T) and older transgenic (TG) coho salmon. Significant differences for pairwise comparisons are indicated by asterisks (*P<0.05, **P<0.01 and ***P<0.001); n.s., non-significant.

Otoliths from NT fish were significantly smaller than those of size-matched transgenic fish in terms of length (OL; −24%; *P*<0.001), width (OH; −19%; *P*<0.001), perimeter (OP; −23%; *P*<0.001) and area (OA; −32%; *P*<0.001) (Table 1). When normalized for standard length, differences in size remained, with normalized otolith length (normalized OL; −24%; *P*<0.001), width (normalized OH; −19%; *P*<0.001), perimeter (normalized OP; −20%; *P*<0.001) and area (normalized OA; −30%; *P*<0.001) significantly smaller in transgenic versus NT fish (Table 1, Fig. 2). We also normalized the otolith morphometric data by eye diameter and found that only the otolith area was significantly reduced (−15%; *P*<0.01) (Tables 1 and 2).

**Fig. 3.**
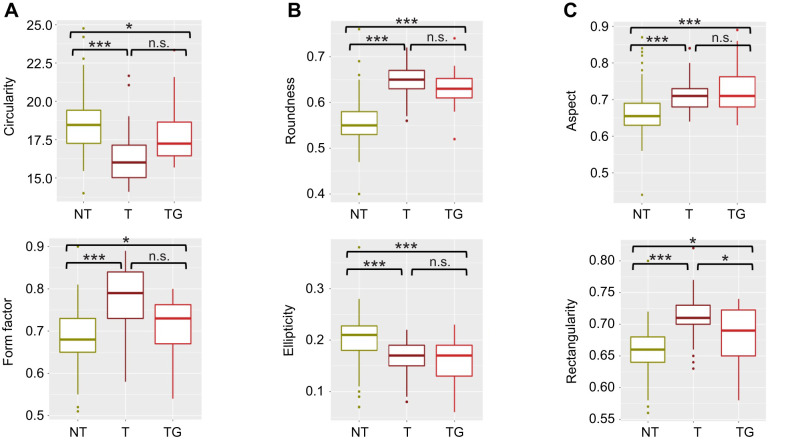
**Otolith shape indexes.** Box and whisker plots for circularity (A), roundness (B), aspect (C), form factor (D), ellipticity (E) and rectangularity (F) for non-transgenic (NT), size-matched transgenic (T) and older transgenic (TG) coho salmon. Significant differences for pairwise comparisons are indicated by asterisks (*P<0.05 and ***P<0.001); n.s., non-significant.

**Fig. 4.**
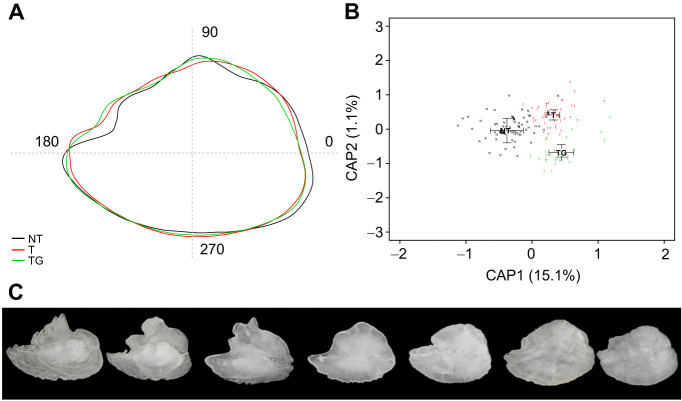
**Otolith shape analysis.** (A) Average otolith shape for non-transgenic (NT), size-matched transgenic (T) and older transgenic (TG) coho salmon. (B) Principal component analysis for NT (black N), transgenic (red T) and older transgenic (green T) fish. The percentage of variability explained by each component (CAP) is indicated in parentheses. (C) Examples of otolith shapes found in the present study.

We also found significant differences when shape indexes were compared, such as a reduction in circularity (−12%; *P*<0.001) and ellipticity (−15.5%; *P*<0.001) in transgenic animals compared with NT fish (Tables 1 and 2, Fig. 3). In contrast, aspect (+6.2%; *P*<0.001), form factor (+13.5%; *P*<0.001), roundness (+15%; *P*<0.001) and rectangularity (+8.3%; *P*<0.001) increased. In order to have a more accurate understanding of the differences in shape, we performed an outline analysis using ShapeR software, which showed that transgenic otoliths were significantly rounder (*P*<0.001) and had a lower individual variability than NT otoliths (Fig. 4).

**Fig. 5.**
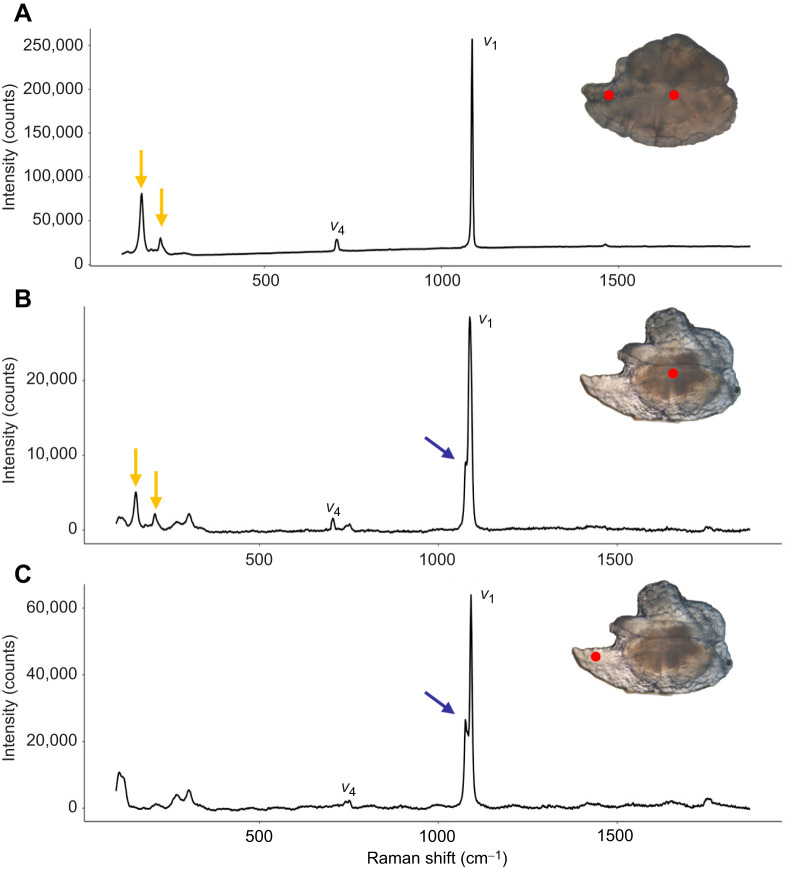
**Raman spectrometry of abnormal calcium carbonate deposition in coho salmon.** Raman spectrometry profiles for transgenic (A) and non-transgenic (B,C) coho salmon otoliths. Typical calcium carbonate *v*_1_ and *v*_4_ peaks are indicated. Typical peaks for aragonite are indicated by yellow arrows, while the typical vaterite double peak in *v*_1_ is indicated by a blue arrow. Insets show representative transgenic and non-transgenic otoliths under light microscopy. The region of the otolith where each spectrum was obtained is marked with a red dot.

**Fig. 6.**
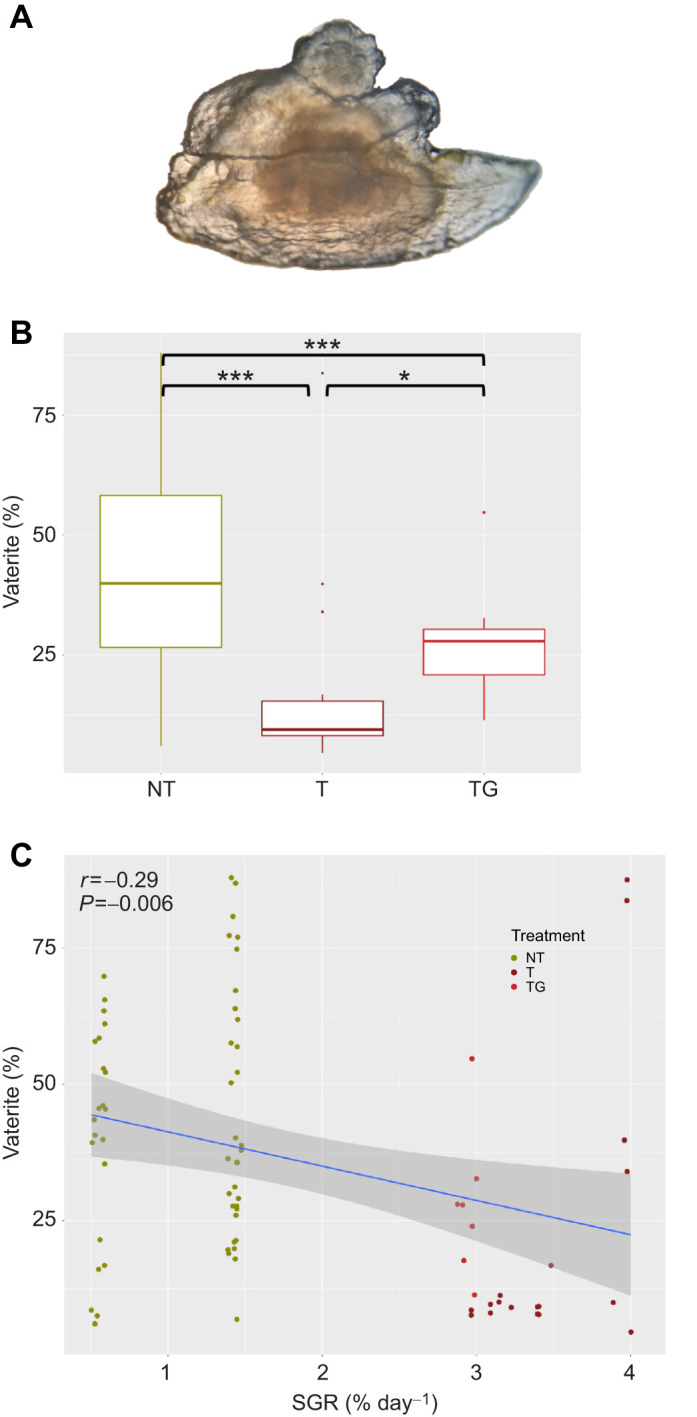
**Vaterite deposition in coho salmon otoliths.** (A) A typical otolith affected by vaterite deposition characterized by a more transparent and non-smooth structure. (B) The percentage of the otolith covered by vaterite for non-transgenic (NT), size-matched transgenic (T) and older transgenic (TG) coho salmon. Significant differences for pairwise comparisons are indicated by asterisks (*P<0.05 and ***P<0.001); n.s., non-significant. (C) Correlation between individual specific growth rates (SGR) and percentage of vaterite present in the otoliths. The shaded area around the linear correlation represents the 95% confidence interval of the correlation. Pearson coefficient of correlation (*r*) and degree of significance (*P*) are indicated.

We also studied the presence of abnormal CaCO_3_ deposition in otoliths and found a significant reduction in the percentage of otoliths showing a clear alteration of their crystallization in transgenic (21–28%) compared with NT (69%) fish (Tables 1 and 2). Their crystal structure and transparency suggested that the abnormal crystal was vaterite; its identification was confirmed by Raman spectrometry (Fig. 5). All otolith readings showed the typical *v*_1_ and *v*_4_ vibrational modes of the CaCO_3_ lattice. Under light microscopy, aragonite appeared as a dense non-translucent mineral (Fig. 5) showing readings associated with this polymorph (two peaks around 205 and 280 cm^−1^). In contrast, vaterite had a transparent appearance (Fig. 5B,C) and showed the characteristic vaterite double peak in the *v*_1_ vibrational mode (Fig. 5B,C). Interestingly, vaterite otoliths always revealed an aragonitic core that, when blasted with the Raman laser, resulted in a mix of aragonite and vaterite readings (Fig. 5B), whereas fully vateritic regions (such as those far from the core; Fig. 5C) showed unequivocal vaterite readings (Sharma et al., 1997). We analysed the proportion of the translucent otolith area in order to determine the percentage of vaterite deposition and found that transgenic fish had a significantly lower percentage of vaterite deposition in the otolith surface (20–26%) than did NT fish (48%; *P*<0.01) (Fig. 6B).

**Fig. 7.**
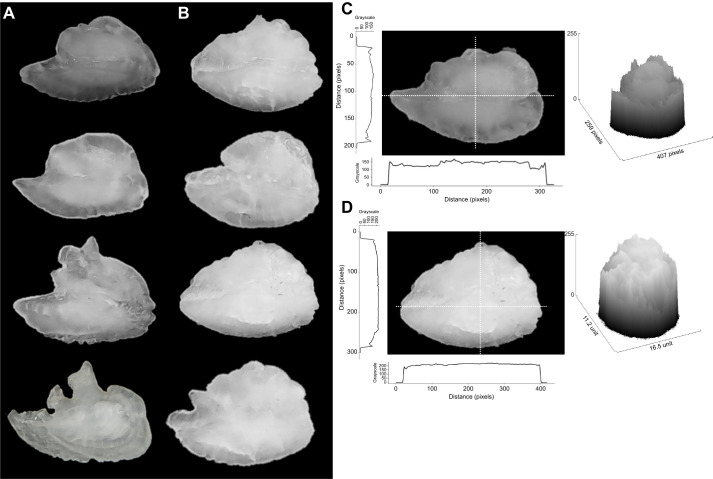
**Otolith opacity of transgenic and non-transgenic coho salmon.** (A) Non-transgenic and (B) size-matched transgenic coho salmon otoliths. (C) Linear grayscale analysis of longitudinal (*y*-axis) and transverse (*x*-axis) sections of non-transgenic and transgenic otoliths. (D) Surface plot of the grayscale values, showing the grayscale colour in the different regions of the otoliths. In all grayscale analysis, some black background was included to better visualize the start and end of the analysis (black background is indicated as a line at 0 level in the grayscale).

We also found that otoliths from the transgenic animals were whiter and more opaque, as confirmed by the colour analysis of the otoliths when transformed to grayscale (Fig. 7). Most NT otoliths had a grayscale average intensity of around 150 (ImageJ scale), but because of some variation within the otoliths, some regions could be darker and less opaque (generally at the edges), with regions closer to 150 being generally at the centre of the otolith (Fig. 7C), indicating a darker average colour. Transgenic otoliths, in contrast, had a grayscale intensity around 200, with much less variation between regions (more homogeneous), indicating a considerably whiter overall colour (Fig. 7C).

## DISCUSSION

Significant differences in shape and composition between otoliths from NT and transgenic fish of matching sizes were observed. However, because of intrinsic differences in specific growth rates found in the present and previous works (Devlin et al., 2004), the animals used were necessarily of different ages (transgenic fish were 213–240 days post-fertilization while NT fish were all >500 days post-fertilization when sampled), and this should be taken into consideration when interpreting some of the results. Our data showed that transgenic animals had a significantly higher specific growth rate (3.57±0.38% day^−1^) compared with NT animals (1.10±0.42% day^−1^; *P*<0.001), as previously reported by other studies (Tables 1 and 2) (Devlin et al., 2004). Despite the two groups of animals being of matching sizes, we found that transgenic fish had much smaller eyes (30% smaller on average). This eye diameter reduction has been observed in previous studies, but no clear explanation for this was given (Devlin et al., 2012; Gaffney et al., 2020; 2018). There is only one previous study in fish that found uncoupled growth rates between the eyes and the rest of the body, with the eyes growing faster than the rest of the body under fasting conditions (Pankhurst and Montgomery, 1994); however, the opposite response was observed in transgenic fish, with the eyes seeming to grow slower than the body. Indeed, when the growth of GH-transgenic salmon is restricted to a normal growth rate, the reduction of eye size does not occur, suggesting the effects may be indirect consequences of growth rate (Devlin et al., 2012). One possible explanation may be related to smaller brains in transgenic fish. For instance, studies in guppies (Corral-López et al., 2017; Näslund, 2014) and killifish (Howell et al., 2021) have correlated eye size with brain size, and some authors have suggested measuring the eye size as a method to estimate gross brain size (Näslund, 2014). Therefore, it is possible that transgenic fish might have smaller brains as a result of a lower expression of *igf1* in their heads as observed in previous work (Bradford and Geen, 1992; Devlin et al., 2012).

Because of the close connection between brain and otolith size, and the very strong correlation we found between otolith size and eye diameter (*r*=0.76; *P*<0.001) in transgenic fish, we tested the effect of normalizing the morphometric data by eye diameter instead of standard length (as is normally done). When this approach was used, otoliths from transgenic fish were only significantly smaller for area (−14%; *P*<0.01) compared with NT fish (Tables 1 and 2). Eye diameter and otolith size were well correlated in transgenic fish, which might indicate that under some specific circumstances, normalization by eye diameter might complement the most classical normalization by length.

Compared with earlier work on *O. kisutch* otoliths (Sweeting et al., 2004) and other species of the genus *Oncorhynchus*, such as Chinook salmon (*O. tshawytscha*) (Oxman et al., 2007) and rainbow trout (*O. mykiss*) (Donohoe et al., 2008), the transgenic animals in the present study had otoliths with an outline similar to that found in wild populations rather than cultured fish. We also considered the effect of age on the differences observed as several studies have shown that age influences otolith shape (e.g. Mapp et al., 2017) (in the present study, to obtain animals of matched size, transgenic and NT animals were necessarily of different ages). To estimate the influence of age and, at the same time, brain size (as estimated by eye diameter), we assessed otolith dimensions and morphology in older transgenic animals (TG group) which were grown to a size (335 days post-fertilization) that matched the eye diameter observed in NT fish at the time of sampling. Although these animals were ∼500% heavier and 67% longer than NT fish (Table 1), the eye diameter was quite similar (+9.8% bigger; *P*=0.28) (Tables 1 and 2). Despite eye diameter being similar between NT and older transgenic fish, the eye in older transgenic individuals represented 14% of the total head size compared with 23% in the NT group. Interestingly, non-normalized OL, OH and OP values in older transgenic fish were similar to those in NT animals (Table 1, Fig. 2). When normalized by eye diameter, only the area of the otoliths from older transgenic fish appeared to be significantly different compared with NT fish (+13%; *P*<0.01).

Again, the disparity between eye size and standard length when normalizing the data indicates that, in some cases, other normalization methods should be considered in combination with animal length. Most interestingly, while the shape of the otoliths from the older transgenic fish was slightly different from that of the younger transgenic animals (indicating some influence of age), the otoliths were still significantly rounder than those from NT fish (Fig. 3,Tables 1 and 2). Furthermore, otolith outlines were much more like those of transgenic than NT fish (Fig. 4A) as can be inferred by the similar position of transgenic and older transgenic groups on component 1 of the outline's principal components analysis (representing 15% of the total variation) (Fig. 4B). Therefore, our analysis of the older transgenic group indicates that neither age nor eye size (as a possible proxy for brain size) seems to have a strong effect on otolith shape.

Vaterite deposition in NT otoliths would explain the differences in shape observed, as vateritic otoliths grow faster and more amorphous than aragonitic ones. Based on research conducted previously which suggested that high growth rates promote vaterite deposition and influence otolith formation (Reimer et al., 2017; Miller and Hurst, 2020), we would expect a higher, or at least similar, incidence of vaterite deposition. However, what we found was a negative correlation between specific growth rate and percentage of vaterite in the otolith (Fig. 6C). It might be suggested that reduced brain size (as a possibility indicated by smaller eye size) in transgenic coho salmon might prevent vaterite deposition by physically constraining otolith growth. While it is true that eye (and possibly brain) and somatic growth are uncoupled, older transgenic animals had a slightly bigger eye diameter (+9.8% increase) compared with NT fish, and as such it would be expected that they should at least have similar vaterite deposition to NT fish, but this was not the case. Another possibility affecting otolith development could be the endocrine peculiarities of GH overexpression. Previous studies have demonstrated the role of thyroid hormones on the promotion of otolith growth and mineralization in salmonids (Melamed et al., 1995; Moav and McKeown, 1992; Schreiber et al., 2010; Shiao and Hwang, 2004), together with its more traditional roles associated with metabolism regulation (Dearl and Volkoff, 2020). Previous work on transgenic coho salmon has shown that T3 levels are elevated in GH-transgenic fish plasma, probably as a result of a reduced hepatic degradation of the T3 in those animals (Eales et al., 2004; Raven et al., 2008). It is possible that the elevated T3 levels could promote a higher mineralization of transgenic fish otoliths, preventing vaterite deposition. It is also possible that GH itself might be contributing to a higher mineralization rate (as suggested by Shinobu and Mugiya, 1995) and preventing vaterite deposition. In the present study, we did not evaluate the mineralization of the otoliths; therefore, further experiments are needed to address this hypothesis. However, we found that otoliths from the transgenic animals were whiter and more opaque, as confirmed by the colour analysis of the otoliths when transformed to grayscale (Fig. 7). Despite this clear trend, an evaluation of the mineralization rate would be revealing to assess the mechanism influencing otolith development in strains of salmon with modified growth physiology.

Another mechanism affecting otolith development (that does not necessarily rule out the effect of GH on mineralization rate) could be that the continuous signal of the hormone may modify the protein matrix of the otoliths. In the last decade, extensive research has been conducted on the role of the protein matrix in otolith mineralization and determination of the main CaCO_3_ polymorph, such as Omp-1, Otoc1, Omm-64, Stm, Stm-l or Sparc (see review by Thomas et al., 2019). Some studies have demonstrated that changes in the expression of *omm-64* and *stm* can modify aragonite precipitation to other polymorphs (Kalka et al., 2019; Poznar et al., 2020) and the imbalance of this protein was the hypothesis suggested by Reimer et al. (2017) to explain why fast-growing fish might replace aragonite with vaterite, a possibility that warrants empirical assessment relating to protein ratios.

Further, other studies have suggested that modifications in the degree of protein matrix phosphorylation can also change the process of mineralization because of the importance of the interactions between the negative charges of the phosphorous group and the positive charges of the CaCO_3_. GH has demonstrated its capacity to modify protein balance in different fish tissues by inducing protein synthesis and phosphorylation either directly (Kittilson et al., 2011; Blasco et al., 2021) or via IGF1 (Reindl et al., 2011; Fuentes et al., 2013). Similarly, it is well known that the thyroid hormones also modify protein balance (Dearl and Volkoff, 2020) and therefore they might also modify the protein composition of the otolith organic matrix, reducing the chances of a shift between aragonite and vaterite. In the present work, we did not analyse otolith protein composition, but potentially the GH or thyroid hormones (directly or indirectly) could modify the protein composition or the phosphorylation degree of the otolith's protein matrix, increasing either one or both processes and reducing the incidence of vaterite.

### Conclusions

In the present work, we have studied the effect of GH transgenesis on the formation of coho salmon otoliths. We found that GH-transgenic fish favoured a rounder otolith shape, resembling wild salmonid otoliths, potentially as a result of their smaller brains or even a higher carbonate deposition. Our data also show that GH-transgenic coho salmon had lower vaterite incidence than NT fish, with the possibility of a higher mineralization due to endocrine signals. A better understanding of how GH might control otolith mineralization and prevent vaterite deposition has important implications for fish welfare.
